# Effectiveness of the Association of Fibrin Scaffolds, Nanohydroxyapatite, and Photobiomodulation with Simultaneous Low-Level Red and Infrared Lasers in Bone Repair

**DOI:** 10.3390/ma17174351

**Published:** 2024-09-03

**Authors:** Jéssica de Oliveira Rossi, Emilie Maria Cabral Araujo, Maria Eduarda Côrtes Camargo, Rui Seabra Ferreira Junior, Benedito Barraviera, Maria Angélica Miglino, Dayane Maria Braz Nogueira, Carlos Henrique Bertoni Reis, Guilherme Eugênio Gil, Thaís Rissato Vinholo, Thiago Pereira Soares, Rogerio Leone Buchaim, Daniela Vieira Buchaim

**Affiliations:** 1 Occupational Medicine, Marilia School of Medicine (FAMEMA), Marilia 17519-030, Brazil; jessica.or@usp.br; 2 Department of Biological Sciences, Bauru School of Dentistry (FOB/USP), University of São Paulo, Bauru 17012-901, Brazil; emi_maria_28@usp.br (E.M.C.A.); rogerio@fob.usp.br (R.L.B.); 3 Medical School, University of Marilia (UNIMAR), Marilia 17525-902, Brazil; maducortes008@gmail.com (M.E.C.C.); guilherme-rf@hotmail.com (G.E.G.); thaisrissato18@gmail.com (T.R.V.); thiagosoaress@gmail.com (T.P.S.); 4 Center for the Study of Venoms and Venomous Animals (CEVAP), São Paulo State University (University Estadual Paulista, UNESP), Botucatu 18610-307, Brazil; rui.seabra@unesp.br (R.S.F.J.); benedito.barraviera@unesp.br (B.B.); 5 Graduate Programs in Tropical Diseases and Clinical Research, Botucatu Medical School (FMB), São Paulo State University (UNESP–University Estadual Paulista), Botucatu 18618-687, Brazil; 6 Postgraduate Program in Structural and Functional Interactions in Rehabilitation, Postgraduate Department, University of Marilia (UNIMAR), Marilia 17525-902, Brazil; miglino@usp.br (M.A.M.); dr.carloshenriquereis@usp.br (C.H.B.R.); 7 Postgraduate Program in Animal Health, Production and Environment, University of Marilia (UNIMAR), Marilia 17525-902, Brazil; 8 Department of Anatomy, Faculty of Higher Education of the Interior of São Paulo (FAIP), Marilia 17512-130, Brazil; dayanenogueira@usp.br; 9 Graduate Program in Anatomy of Domestic and Wild Animals, Faculty of Veterinary Medicine and Animal Science, University of São Paulo (FMVZ/USP), Sao Paulo 05508-270, Brazil; 10 Medical School, University Center of Adamantina (UNIFAI), Adamantina 17800-000, Brazil

**Keywords:** bone regeneration, low-level laser therapy, bone substitutes, fibrin biopolymer, fibrin sealant, hydroxyapatite, photobiomodulation, lasers, regenerative medicine

## Abstract

Biomaterials and biopharmaceuticals for correcting large bone defects are a potential area of translational science. A new bioproduct, purified from snake venom and fibrinogen from buffalo blood, aroused interest in the repair of venous ulcers. Expanding potential uses, it has also been used to form biocomplexes in combination with bone grafts, associated with physical therapies or used alone. The aim of this preclinical study was to evaluate low-level laser photobiomodulation (PBM) in critical defects in the calvaria of rats filled with nanohydroxyapatite (NH) associated with the heterologous fibrin biopolymer (HFB). Sixty animals were used, divided into six groups (*n* = 10 each): G1 (NH); G2 (HFB); G3 (NH + HFB); G4 (NH + PBM); G5 (HFB + PBM); G6 (NH + HFB + PBM). PBM simultaneously used red (R) and infrared (IR) light emission, applied intraoperatively and twice a week, until the end of the experiment at 42 days. Microtomography, bone formation can be seen initially at the margins of the defect, more evident in G5. Microscopically, bone formation demonstrated immature and disorganized trabeculation at 14 days, with remnants of grafting materials. At 42 days, the percentage of new bone formed was higher in all groups, especially in G5 (HFB, 45.4 ± 3.82), with collagen fibers at a higher degree of maturation and yellowish-green color in the birefringence analysis with Picrosirius-red. Therefore, it is concluded that the HFB + PBM combination showed greater effectiveness in the repair process and presents potential for future clinical studies.

## 1. Introduction

The resolution of larger bone defects poses a challenge for regenerative science, both in medical and dental fields. Current treatment options are complex, considering the morbidity and incidence of late complications [1,2]. The bone architecture can be compromised by trauma, congenital anomalies, and degenerative diseases. Therefore, tissue engineering and regenerative medicine strive to develop reconstructive therapies aimed at morphophysiological restoring damaged tissue [3].

There are several therapies available, but autogenous grafts are still considered the gold standard, providing the best tissue repair process by combining regenerative properties in terms of osteogenesis, osteoconduction, and osteoinduction. However, there are limiting factors, including the need for two surgical sites, problems related to local morbidity, and limited quantity, which drives new studies in the production and improvement of new bone substitutes [4].

Bioceramics are an important class of biomaterials, named for their ceramic origin [5]. They possess properties such as low density, chemical stability, high wear resistance, and biocompatibility, the latter being a direct result of their compositions containing ions typically found in the physiological environment, such as Ca^2+^, K^+^, Mg^2+^, and Na^+^ [6,7]. Notable bioceramic materials include hydroxyapatite, alumina, zirconia, and bioglass [8].

Bioceramics are classified according to their interaction with tissues as bioinert or bioactive, with the latter further subdivided into resorbable or non-resorbable [9,10]. Bioinert materials do not form interfacial biochemical bonds with the tissues they contact, having almost no influence on them, as seen with alumina and zirconia. In contrast, bioactive materials have this interfacial bonding ability with bone tissue, as is the case with hydroxyapatite and bioglass [11,12].

Hydroxyapatite (HAp) is a calcium phosphate with the chemical formula Ca_10_(PO_4_)_6_(OH)_2_. The stoichiometry of HAp is of great significance, as slight imbalances in the Ca/P ratio can lead to the formation of other phases that affect the material’s interaction with biological tissues [13]. In addition to biocompatibility, HAp constitutes up to 69% of natural human bone mass and exhibits desirable properties such as osteoconductivity [14], having the ability to provide the appropriate structure for vascularization, cell infiltration, cartilage formation, and bone tissue deposition [15]. It also possesses bioactive properties due to its chemical composition and crystallographic properties, which are similar to the mineral portion of human hard tissues [16,17].

Although this method is clinically established, new therapeutic approaches can be used, such as scaffold association, forming specialized tissue constructs to achieve a synergistic effect and better overall properties, which are the results when compared to conventional grafting techniques [18,19]. This approach seeks to mimic the original bone structure, favoring the attraction of bone-producing cellular elements (osteoblasts), increasing local growth factors and, in this way, contributing to the production and mineralization of new bone tissue [20,21].

Natural biopolymers, such as fibrin sealants, are potential candidates for the combined use with bone grafts [22,23], enabling the fabrication of multifunctional three-dimensional scaffolds that reduce bleeding through hemostatic mechanisms and increase graft stability in the surgical site. This provides greater time of cellular support throughout the bone repair process, improving the local microenvironment, and consequently increasing the success rate of grafting [24].

Most commercially available preparations consist of homologous plasma hemocomponents, which are high-value and carry a risk of viral transmission. Autologous formulations of fibrin sealants or Platelet-Rich Fibrin (PRF) are a treatment option; however, they are unviable for severely injured patients or in unforeseen emergencies [25]. To address this issue, researchers from the Center for the Study of Venoms and Venomous Animals (CEVAP, São Paulo State University, UNESP, Botucatu, Brazil) developed an effective, safe, and affordable version by replacing human fibrinogen with plasma fibrinogen from large animals, *Bubalus bubalis*, and thrombin with serine protease extracted from the venom of *Crotalus durissus terrificus* [26]. Initially, this fibrin sealant was used for the treatment of chronic venous ulcers, peripheral nerve repair, and as an alternative to conventional sutures, among other applications, with promising results [27]. Indeed, the excellent biocompatibility, controllable biodegradability, intrinsic bioactivity, biomimicry, and many other unique characteristics have made this therapeutic formulation viable for association with biomaterials [28].

In the quest to improve outcomes of reconstructive surgical interventions, complementary therapeutic modalities have been used, such as non-invasive low-level laser therapy (LLLT), to accelerate tissue regeneration and modulate inflammatory processes in cells with functional deficits [29]. Photobiomodulation (PBM) has been shown to be effective in modulating biochemical reactions, increasing adenosine triphosphate (ATP) supply, enhancing cell membrane permeability, enabling calcium influx, stimulating cell differentiation and proliferation, regulating growth factors and pro-inflammatory cytokines, inducing synthesis, and remodeling collagen and angiogenesis [30,31].

Considering these issues, the conduct of this study is justified by its novel approach, as follows: the combination of 100% heterologous fibrin biopolymer with a paste biomaterial, nano hydroxyapatite, along with a low-level laser photobiomodulation protocol featuring simultaneous red and infrared laser. Therefore, the goal was to evaluate laser photobiomodulation in critical bone defects on the calvaria of rats filled with nanohydroxyapatite associated with this new formulation of heterologous fibrin biopolymer.

## 2. Materials and Methods

### 2.1. Experimental Protocol

Sixty adult rats (*Rattus norvegicus*), Wistar Hannover strain, male, 90 days old, with a body mass of approximately 320 g, obtained from ANILAB (Laboratory Animals Breeding and Trading Ltd., Paulinia, Brazil), were used. The animals were housed in conventional cages at the Animal Facility of the University of Marilia (UNIMAR, Marilia, Brazil), with ad libitum access to Nuvilab^®^ rodent feed (Curitiba, Brazil) and filtered water, in a climate-controlled environment at 22 °C, with an air exhaust and a 12 h light/dark cycle. The research was approved by the Ethics Committee on Animal Use (CEUA) of the University of Marilia (Protocol No 03/2022).

In this study, the ARRIVE (Animal Research: Report of in vivo Experiments) checklist [32] was used to assess the methodological steps aimed at method replication and result verification. Animals were monitored throughout all experimental phases and were randomly allocated into 6 groups according to the type of defect filling and photobiomodulation therapy.

**Figure 1:**
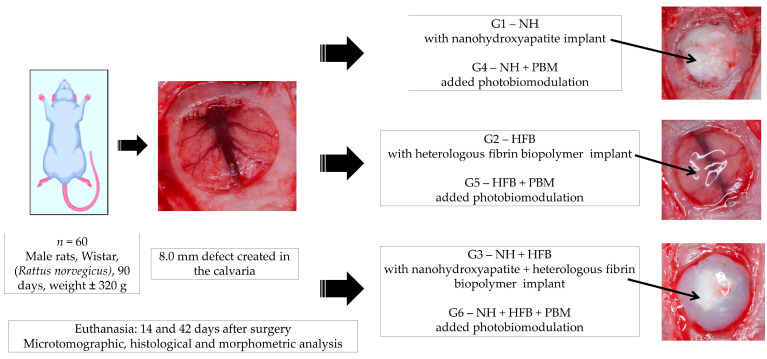
Experimental design: 60 rats randomly distributed into 6 groups (*n* = 10 each) according to the type of defect filling and photobiomodulation treatment: G1, defect filled with nanohydroxyapatite; G2, defect filled with heterologous fibrin biopolymer; G3, defect filled with nanohydroxyapatite and heterologous fibrin biopolymer; G4, defect filled with nanohydroxyapatite and low-level laser photobiomodulation; G5, defect filled with heterologous fibrin biopolymer and photobiomodulation; G6, defect filled with nanohydroxyapatite, heterologous fibrin biopolymer, and photobiomodulation.

The rats were randomly assigned to 6 groups (*n* = 10 each) according to the type of defect filling and photobiomodulation treatment: G1, defect filled with nanohydroxyapatite; G2, defect filled with heterologous fibrin biopolymer; G3, defect filled with nanohydroxyapatite and heterologous fibrin biopolymer; G4, defect filled with nanohydroxyapatite and low-level laser photobiomodulation; G5, defect filled with heterologous fibrin biopolymer and photobiomodulation; G6, defect filled with nanohydroxyapatite, heterologous fibrin biopolymer, and photobiomodulation (Figure 1).

### 2.2. Treatments

#### 2.2.1. Nanocrystalline Hydroxyapatite

The product Bone-Oss^®^ (Bioceramed, São Julião do Tojal, Loures, Portugal), available in 1cc syringes, reference BO011, lot 22n001201, used in this experiment, is an injectable fully resorbable bone substitute composed of nanocrystalline hydroxyapatite (HA) in an aqueous solution (approximate size of 100 nm). It has a total porosity of 60–80% and a pore size ranging from 200 μm to 500 μm. The product is supplied ready-to-use in a needle-free transparent syringe and is indicated by the manufacturer to be used for bone defect filling. It consists of synthetic nanocrystalline ceramic material based on calcium phosphate, exhibiting biological and technical characteristics suitable for bone graft applications, given that calcium orthophosphates are important inorganic constituents of vertebrate hard tissues. For each animal, 0.2 cc of nanohydroxyapatite paste was standardized for the complete filling of the bone defect.

#### 2.2.2. Heterologous Fibrin Biopolymer

The heterologous fibrin biopolymer, initially known as snake-venom-derived fibrin sealant or glue, was kindly provided by the Center for the Study of Venoms and Venomous Animals (CEVAP, São Paulo State University, UNESP, Botucatu, Brazil). The components and application formula are detailed in patent BR 102014011432-7, issued on 6 July 2022, by the Brazilian National Institute of Industrial Property (INPI). It consists of three distinct frozen fractions, which are thawed, mixed, and homogenized prior to use. Fraction 1 contains a thrombin-like protein (gyroxin) obtained from the venom of Crotalus durissus terrificus, which includes calcium chloride as the diluent. Fraction 2 is fibrinogen (cryoprecipitate) derived from the blood of buffalo (Bubalus bubalis). The amount for each fraction in each critical defect in the calvaria was as follows: 20 µL of Fraction 1, 20 µL of diluent, and 40 µL of Fraction 2. The results of the phase I/II non-randomized clinical trial using this heterologous fibrin biopolymer in the treatment of venous ulcers were published in 2021 [33].

#### 2.2.3. Photobiomodulation Low-Level Laser Therapy

Immediately after the surgical procedure, the animals in groups G4, G5, and G6 underwent photobiomodulation therapy until the end of the experiment at 14 and 42 days, using the following protocol (Table 1).

### 2.3. Experimental Surgery and Euthanasia

The surgical procedures were conducted in the surgery laboratory of the Animal Facility at the University of Marilia (UNIMAR, Marilia, Brazil). After weighing, the animals underwent general intraperitoneal anesthesia in the lower left abdominal quadrant. Anesthesia was induced using a combination of 80 mg/kg of body weight of ketamine hydrochloride (Dopalen^®^, Ceva, Paulinia, Brazil) as the sedative and 10 mg/kg of body weight of xylazine hydrochloride (Anasedan^®^, Ceva, Paulinia, Brazil) as the muscle relaxant, with rigorous monitoring throughout the procedure.

Next, the surgical site was shaved and cleansed with a topical solution of Povidone-Iodine (Povidine^®^ Antiseptic, Vic Pharma Ind and Comércio Ltd.a, São Paulo, Brazil) at 10%. A semilunar incision was made in the skin using a no. 15 surgical blade (Embramax^®^, São Paulo, Brazil), and the periosteum was dissected using a periosteal elevator. The tissues were then retracted, exposing the outer surface of the parietal bones.

Using a trephine drill (Neodent^®^, Curitiba, Brazil), a bone defect of 8.0 mm in diameter was created over the sagittal suture on the parietal bones using a dental contra-angle handpiece (Driller^®^, Carapicuiba, Brazil) and an electric micromotor (Driller^®^ BLM 600 Baby, Carapicuiba, Brazil). The rotation speed used was 1500 rpm, with saline irrigation, and care was taken to maintain the integrity of the dura mater and avoid reaching the brain.

The defect treatments were previously described in the experimental protocol (Figure 1). The retracted tissues were repositioned, completely covering the defects, and sutured layer by layer using 4-0 silk suture (Ethicon, Johnson & Johnson Company, São Paulo, Brazil). The surgical area was cleaned with sterile gauze and 2% chlorhexidine topical antiseptic (Riohex^®^ Farmacêutica Rioquímica Ltd.a, São José do Rio Preto, Brazil).

Subsequently, as medication, a single dose of antibiotic Flotril^®^ 2.5% (Schering-Plough, Rio de Janeiro, Brazil) was administered at 0.2 mL/kg, and analgesic Dipirona Analgex V^®^ (Agener União, São Paulo, Brazil) at 0.06 mL/kg, intramuscularly. The analgesic was maintained for 3 days, followed by administration of acetaminophen (Paracetamol Medley^®^, São Paulo, Brazil) at a dose of 200 mg/kg, 6 drops/animal dissolved in the drinking water, until euthanasia.

At 14 and 42 days post-surgery, 5 animals from each group per time point were euthanized with an overdose of general anesthetic (triple dose-240 mg/kg ketamine + 30 mg/kg xylazine). After confirming the animal’s death, the surgical site (cranial vault and soft tissues) was removed and fixed in 10% formalin, phosphate buffer pH 7.2, for 48 h, and subsequently prepared for examination using a micro-computed tomography scanner.

### 2.4. Micro-Computed Tomography (µ-CT)

After fixation of the bone fragments, the specimens underwent X-ray beam scanning using the SkyScan 1272 micro-computed tomography system (Bruker-microCT^®^, Kontich, Belgium) at the Araçatuba School of Dentistry (São Paulo State University, Araçatuba, Brazil). The system operated at 50 kV and 800 µA. The specimens were rotated 360° with a rotation step of 0.5 and an isotropic resolution of 19.6 µm, resulting in an approximate acquisition time of 41 min and 32 s per sample.

The images of each specimen were analyzed and reconstructed using the specific software 64Bits270013 (Bruker^®^, Kontich, Belgium) and the NRecon^®^ Program (version 1.6.8.0, SkyScan, 2011, Bruker-microCT^®^, Kontich, Belgium). The Data Viewer^®^ software version 1.4.4 64-bit (for linear measurements in coronal, transaxial, and sagittal axes) and CTvox^®^ version 2.4.0 r868, Bruker Micro CT, Kontich, Belgium, were used for two-dimensional visualization, followed by qualitative analysis of the newly formed bone tissue.

### 2.5. Histological Processing

After microtomography, the specimens were washed in running water (for 24 h) and subjected to decalcification in a solution of EDTA, containing 4.13% Tritiplex^®^ III (Merck KGaA, Hessen, Germany) and 0.44% sodium hydroxide (Labsynth^®^, São Paulo, Brazil). The solution was changed weekly over a period of approximately 6 weeks. After demineralization, the specimens were dehydrated in an increasing series of ethanol, clarified in xylene, and embedded in Histosec^®^ processed paraffin (Merck, Hessen, Germany). Coronal histological sections, semi-serial, were then performed at the central region of the defect using a semi-automatic microtome Leica^®^ RM2245 (Leica Biosystems, Wetzlar, Germany), with a thickness of 5 µm for hematoxylin-eosin staining, Masson’s trichrome, and Picrosirius-red.

### 2.6. Histomorphological and Histomorphometric Analysis

The entire extent of the defect was considered for histomorphological description, analyzing granulation tissue, inflammatory infiltrate, and newly formed tissue in each defect. Four semi-serial sections of each defect were analyzed using an Olympus^®^ BX50 light microscope (Olympus Corporation, Tokyo, Japan), and images were captured with the attached digital camera (Olympus DP 71^®^, Tokyo, Japan) using the image capture software DP Controller 3.2.1.276 (2001–2006, Olympus Corporation^®^, Tokyo, Japan).

The sections stained with Picrosirius-red for collagen birefringence analysis were evaluated under polarized light using a high-resolution digital camera Leica DFC 310FX (Leica^®^, Microsystems, Wetzlar, Germany) connected to the Leica DM IRBE confocal laser microscope and LAS capture system (Leica Microsystems^®^, version 4.0, Heerbrugg, Switzerland).

Subsequently, for quantification of newly formed bone (as a percentage, %), the defect was evaluated using the image analysis program AxioVision software (version 4.8, Zeiss Microsystems^®^ GmbH, Jena, Germany) to determine the total analyzed area (A) and the area occupied by each constituent in the defect (Ai) in PIXEL units. The volume density (Vvi) of each type of structure was calculated using the relationship: Vvi = (Ai/A)*100.

### 2.7. Statistical Analysis

The quantitative histomorphometric results were subjected to normality testing (Kolmogorov–Smirnov) and homoscedasticity testing (Bartlett). Subsequently, analysis of variance (ANOVA) was performed, and means were compared using Tukey’s test to analyze groups within each period separately. An unpaired Student’s *t*-test was used to compare each group between 14 vs. 42 days. Statistical analysis was conducted using GraphPad Prism (GraphPad^®^ Software version 8.0, La Jolla, CA, USA), with a significance level of 5%.

## 3. Results and Discussion

### 3.1. Microcomputed Tomography (µ-CT)

In the 14-day period, the two-dimensional transaxial microtomographic images revealed an irregular pattern of bone formation in all groups. This process was characterized by the gradual increase in bone tissue density in the peripheral regions of the bone defect, highlighted by the intensification of gray shades in the images. Residual nano hydroxyapatite was also present, dispersed in the surgical cavity. In dental practice, this imaging method is widely used for the evaluation of alveolar bone loss and the analysis of the effects of some medications that alter bone architecture, such as zoledronate [34,35]. Additionally, microtomography is also used in the analysis phases of hydroxyapatite-based biomaterials in their physicochemical characterization [36].

**Figure 2:**
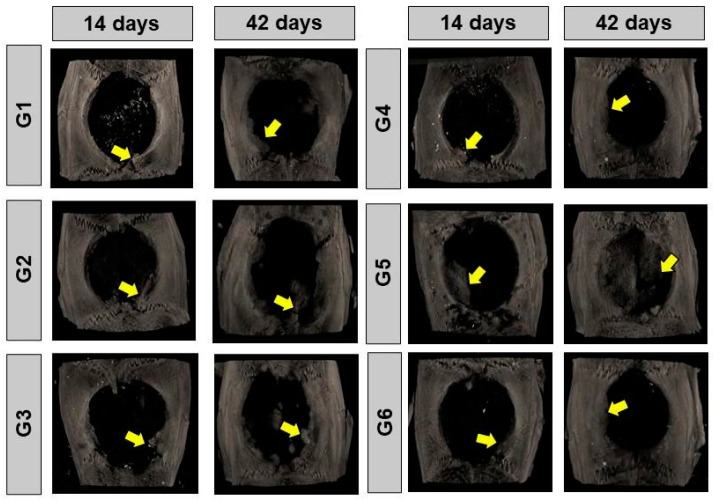
Reconstructed two-dimensional microtomographic images (2D) of the bone defects in rat calvariae at 14 and 42 days, respectively. G1, defect filled with NH; G2, defect filled with HFB; G3, defect filled with NH + HFB; G4, defect filled with NH + PBM; G5, defect filled with HFB + PBM; G6, defect filled with NH + HFB + PBM. Yellow arrows indicate new bone formed in a centripetal manner at the edges of the defect.

At 42 days, a qualitative increase in new bone growth was observed. However, the defect was still not filled and, in most groups, remained restricted to the surgical edges, with localized areas of mineralized tissue. The bone formation process was visibly enhanced using the fibrin biopolymer in G2 and G5. Additionally, PBM therapy also contributed to improving new bone formation (Figure 2). To evaluate the bone formed after treatments, microtomography can be used as one of the quantification methods [37], but in our experimental protocol, we preferred to perform histomorphometry on the histological slides obtained after decalcification of the calvariae, as in previous studies conducted by our research group [38].

### 3.2. Histomorphology

**Figure 3:**
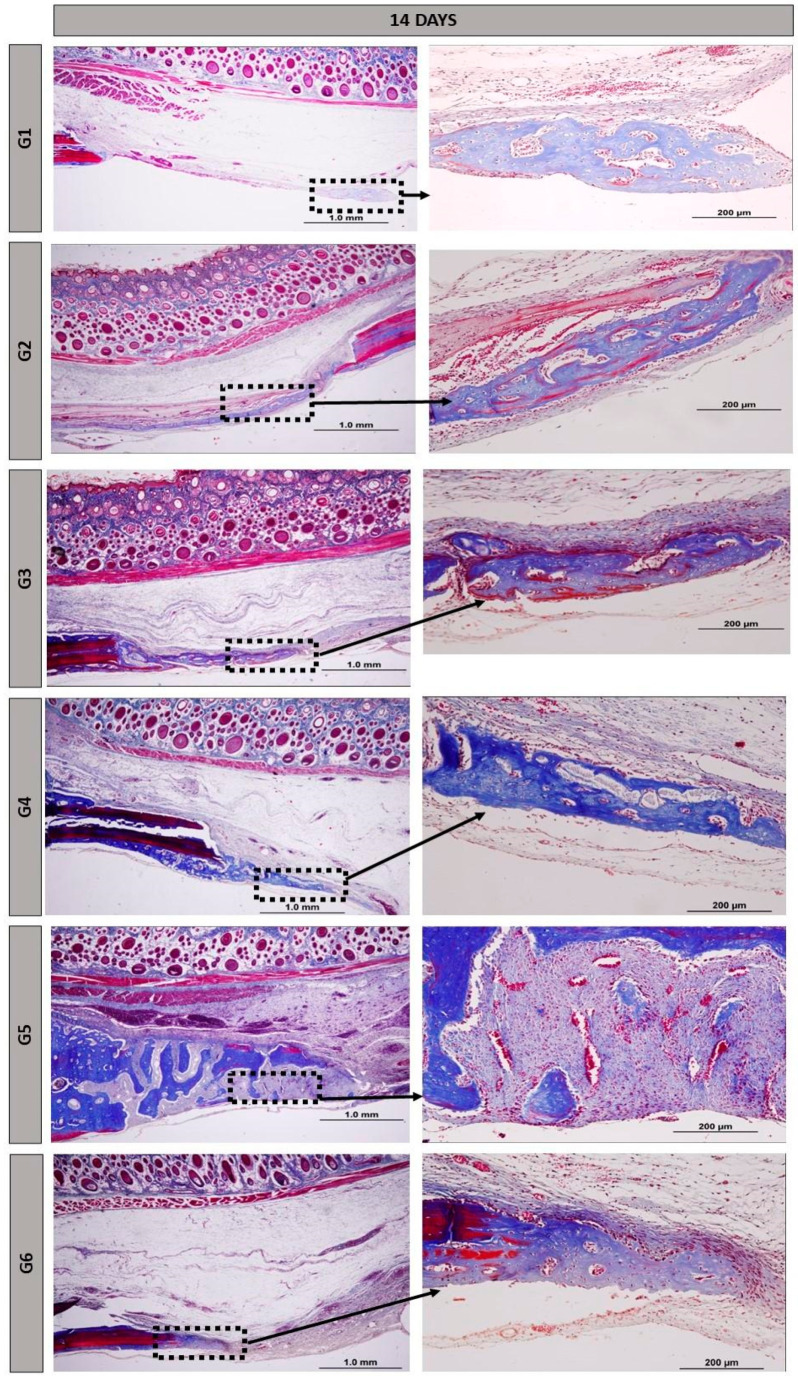
Histological images of the surgical cavity at 14 days post-operation, stained with Masson’s trichrome, in the following groups: G1, defect filled with NH; G2, defect filled with HFB; G3, defect filled with NH + HFB; G4, defect filled with NH + PBM; G5, defect filled with HFB + PBM; G6, defect filled with NH + HFB + PBM. Magnifications of 4× and 20×.

At 14 days, all groups exhibited distinct characteristics, with the defect being filled with reactive connective tissue, rich in cells and permeated by inflammatory cells. Random arrangements of thin collagen fibers and remnants of the NH and HFB biomaterials were noticeable (Figure 3).

**Figure 4:**
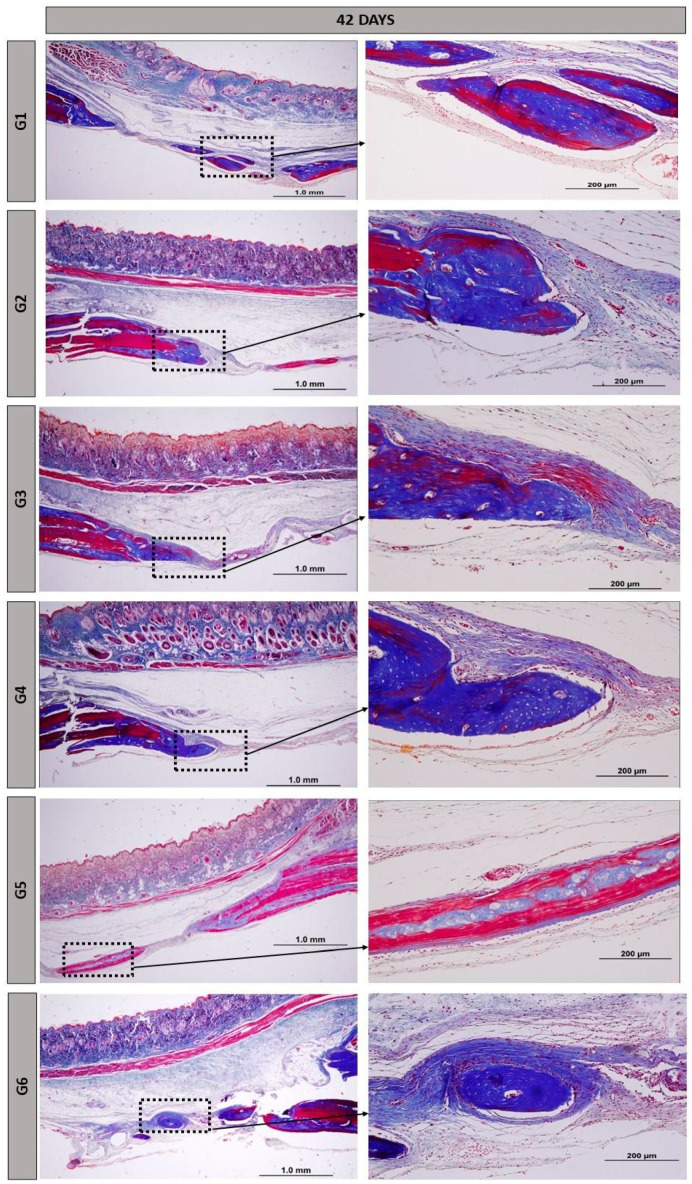
Histological images of the surgical cavity at 42 days post-operation, stained with Masson’s trichrome, in the following groups: G1, defect filled with NH; G2, defect filled with HFB; G3, defect filled with NH + HFB; G4, defect filled with NH + PBM; G5, defect filled with HFB + PBM; G6, defect filled with NH + HFB + PBM. Magnifications of 4× and 20×.

At the 42-day period, the formation of newly generated bone remained primarily confined to the margins of the defect, but was more pronounced than at 14 days. There was a progressive resorption of the biomaterials accompanied by an increase in bone tissue at the edges of the defect and on the surface of the particles found in the central areas of the defect in group G5 (Figure 4).

The height of the remaining bone in the surgical region was preserved in all bone defects. New bone tissue continued to grow, but remained confined to the edges of the defects, with areas of focal mineralization occurring around the nano hydroxyapatite particles. Group G5 showed more advanced maturation, with more organized and mature bone areas forming concentric lamellae surrounded by regions of immature bone trabeculae. Our findings are consistent with other similar studies in the field, demonstrating that the use of biopolymer, sealant, or other fibrin derivatives aids in maintaining the height and thickness of cranial bone [39], dental alveoli [40], or long bones [41].

At 14 days, the presence of vascular buds was observed, mainly in groups G5 and G6, possibly because of PBM therapy on local microcirculation. Photobiomodulation plays an important role in tissue regeneration due to its direct beneficial effects at the irradiated site, including promoting angiogenesis, the formation of new blood vessels. The budding of specialized blood vessels contributes to vascularized bone, which is essential for tissue bioengineering [42].

Nanohydroxyapatite demonstrated a biological response without intense inflammatory reactions; the particles were not encapsulated or rejected at the implantation site and allowed for the adjacent osteoprogenitor cells to differentiate through the structure generated by these materials, consistent with osteoconduction. The morphology of nanoparticles, regardless of their charge or chemistry, influences the induction of inflammation, especially in the acute phase [43].

As surgical cavities where the heterologous fibrin biopolymer was implanted alone, G2 and G5 showed marked angiogenesis starting from 14 days, along with the presence of reactive tissue and the maintenance of vascular spaces after 42 days. Fibrin matrices with Vascular Endothelial Growth Factor (VEGF) provide support for all stages of new blood vessel sprouting, from endothelial cell migration to their maturation [44]. Bone formation was more intense not only at the defect margins, but also in areas where bone exhibited characteristics of organization and maturation (Images 3 and 4). The 6-week period, or 42 days, is well-established in the literature for evaluating post-traumatic bone formation in rats [45,46,47].

**Figure 5:**
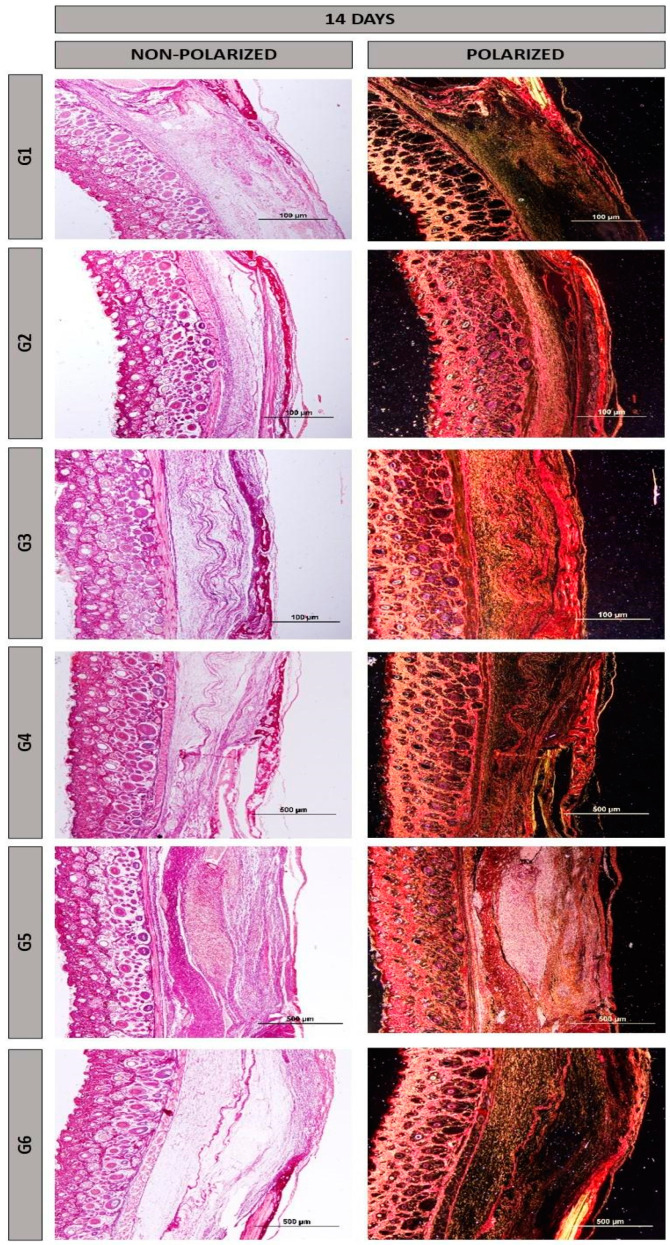
Histological images of the surgical cavity at 14 days post-operation, stained with Picrosirius-red with or without polarization, in the following groups: G1, defect filled with NH; G2, defect filled with HFB; G3, defect filled with NH + HFB; G4, defect filled with NH + PBM; G5, defect filled with HFB + PBM; G6, defect filled with NH + HFB + PBM. Magnification of 10×.

**Figure 6:**
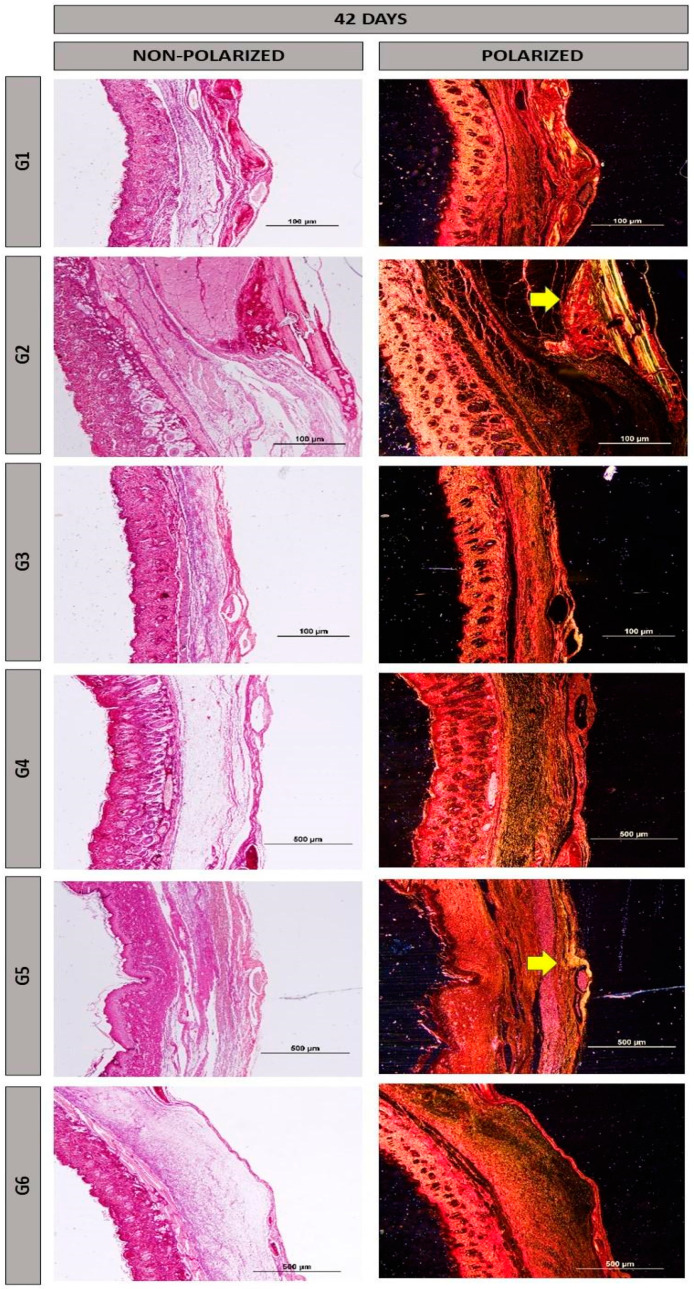
Histological images of the surgical cavity at 42 days post-operation, stained with Picrosirius-red with or without polarization, in the following groups: G1, defect filled with NH; G2, defect filled with HFB; G3, defect filled with NH + HFB; G4, defect filled with NH + PBM; G5, defect filled with HFB + PBM; G6, defect filled with NH + HFB + PBM. Magnification of 10×. Yellow arrows highlight regions with yellowish collagen fibers, indicating higher maturation.

In the birefringence of collagen fibers stained with Picrosirius-red under polarization, reddish colors were observed mainly at 14 days (Figure 5), with a tendency towards maturation at 42 days (Figure 6), displaying yellow and green hues. Additionally, there was an organization of the fibrillar arrangement, especially in groups where the heterologous fibrin biopolymer was implanted alone, without association with nanohydroxyapatite (groups G2 and G5, indicated by yellow arrows). The fine collagen fibers stained more intensely with Picrosirius-red, and their visualization was enhanced using polarization [48,49], facilitating their analysis of structural characteristics.

### 3.3. Histomorphometry

On the slides stained with hematoxylin–eosin, measurements of newly formed bone (percentage, %) were performed. At 14 days, there was a significant quantitative difference, with group G5 showing the highest percentage of bone (22.38 ± 4.75; mean ± standard deviation) compared to the other groups. There was no significant difference between groups G1, G4, and G6, but there was a difference compared to groups G2 and G3.

**Figure 7:**
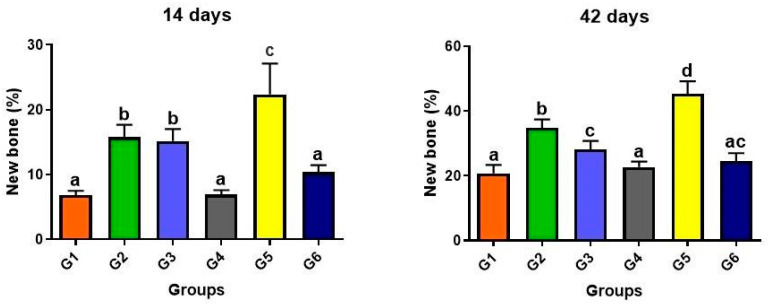
Graphs showing the percentage of new bone formed within the surgical cavity in the groups evaluated at 14 and 42 days post-surgery. Different lowercase letters (a ≠ b ≠ c ≠ d) indicate statistically significant differences (*p* < 0.05). Analysis of variance (ANOVA) and means were compared using Tukey’s test. Groups: G1, defect filled with NH; G2, defect filled with HFB; G3, defect filled with NH + HFB; G4, defect filled with NH + PBM; G5, defect filled with HFB + PBM; G6, defect filled with NH + HFB + PBM.

At 42 days, the highest percentage of bone formation also occurred in group G5 (45.4 ± 3.82), with a significant difference compared to the other groups. There was no significant difference statistically between groups G1, G4, and G6. However, there was a significant difference between groups G2 and G3 (Figure 7).

The association of fibrin matrices with other biomaterials represents a promising alternative in tissue bioengineering. These biocomplexes can combine therapeutic advantages from each biomaterial used, with their three-dimensional meshes serving as a scaffold for cellular and vascular elements. Examples include fibrin-rich biomaterials such as platelet-rich fibrin (PRF), platelet-rich plasma (PRP), leukocyte-platelet-rich fibrin (L-PRF), sealants, or fibrin biopolymers. These combinations enhance the bone repair process [50,51,52].

**Figure 8:**
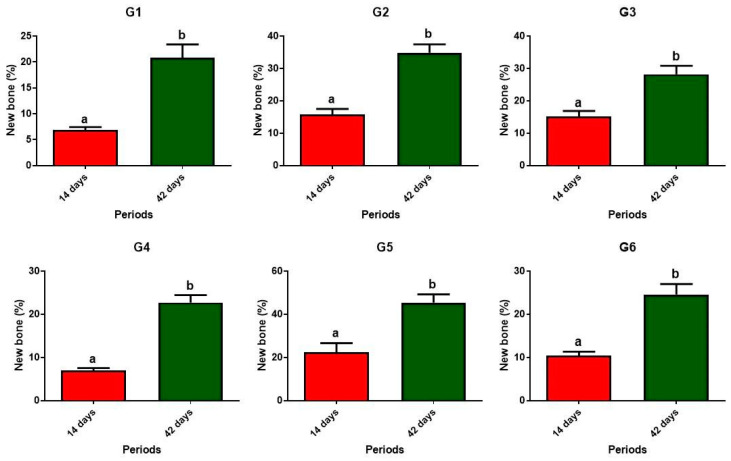
Graphs depicting the percentage of new bone formed within the surgical cavity in the evaluated groups, comparing 14 versus 42 days post-surgery. Different lowercase letters (a ≠ b) indicate statistically significant differences (*p* < 0.05). Unpaired Student’s *t*-test. Groups: G1, defect filled with NH; G2, defect filled with HFB; G3, defect filled with NH + HFB; G4, defect filled with NH + PBM; G5, defect filled with HFB + PBM; G6, defect filled with NH + HFB + PBM.

When comparing each individual group, during the 14 and 42-day bone formation periods, significant differences were observed in all cases, with the higher percentage always occurring at 42 days (Figure 8).

Based on the quantitative results, it is noticeable that groups G2 and G5 performed better, having been filled solely with fibrin biopolymer, with or without photobiomodulation, respectively, in both evaluated periods of 14 and 42 days. This association provides conditions for significant cellular and vascular proliferation, particularly of bone growth factors and osteoprogenitor cells [53,54]. It also supports preventing the invasion of soft tissues into the defect by accelerating new bone formation, acting as a barrier like guided tissue regeneration (GTR) procedures that require the use of resorbable or non-resorbable membranes [55,56]. The association of HFB with particulate biomaterials and PBM, in previous studies by our group [21,23,38,57], has proven to be effective for bone repair. Perhaps the paste presentation of NH used in this study is not ideal for addition to HFB, creating a biocomplex that may have delayed cellular and vascular proliferation into the defect.

There are mechanisms that indicate that a PBM with a low-level laser enhances the healing of bone defects, especially those of critical dimensions, mainly by stimulating local microcirculation, creating conditions in the microenvironment to attract osteoprogenitor cells [57]. A recent clinical study demonstrated that the use of a 940 nm infrared laser in the alveoli of extracted teeth demonstrated that L-PRF (fibrin rich in leukocytes and platelets) was effective for healing in the short-term, while PBM collaborates mainly in the long-term, with better formation and organization of bone trabeculae [58]. Therefore, contextualizing our findings in general with the literature in the area, photobiomodulation using LLLT improves the formation of new bone and local mineralization, accelerating morphological and functional rehabilitation, and consequently reducing the problems generated by bone lesions [59].

In isolation, fibrin scaffolds, through the conversion of fibrinogen into a three-dimensional fibrin network, promote the release of growth factors, with the purpose of promoting vascular formation and proliferation, as well as chemotaxis of cellular elements [60]. In turn, hydroxyapatite is highly biocompatible and osteoconductive, providing support for the growth of new bone tissue [61], and PBM increases the biological response of bone tissue by modulating the inflammation process and stimulating genes that provide early recruitment of osteoprogenitor cells that are related to bone repair [62]. The combined use of therapies and biomaterials, as used in this experimental protocol, may present synergistic effects, in which the isolated properties of each one can be enhanced by their associations. The molecular mechanisms by which LLLT, with its wavelengths, triggers cell proliferation and differentiation are not fully known. One of the hypotheses is that by acting on mitochondria and through Reactive Oxygen Species (ROS), which can activate several molecular pathways and signaling cascades, it causes the activation of several transcriptional factors [63]. In tissue regeneration, the functional design of scaffolds is very important for cell migration and growth. A rabbit study on corneal injury repair revealed that the performance of biomaterials depends mainly on the level of hyaluronan oxidation and that a high degree of oxidation of microcarriers promotes keratocyte biosynthesis [64].

Regarding the molecular mechanisms of cell proliferation, we can also consider that cytochrome c oxidase is biostimulated with the use of LLLT in the wavelength bands of 620 nm, 680 nm, 760 nm, and 825 nm. However, its highest stimulation peak occurs around 760–780 nm. As used in our preclinical study, in the red and infrared laser wavelengths, the cytochrome c oxidase (COI) enzyme is the primary receptor, stimulating complex IV of the mitochondrial chain in the production of ATP (adenosine triphosphate) for the cells. Therefore, with the activation of this photoreceptor, the levels of respiration, ATP, and ROS are increased, causing these biochemical changes to increase cell proliferation and contribute to accelerating the tissue repair process [65,66,67].

The different laser therapy protocols, given all of the parameters used and each type of equipment, generate a comparative difficulty in local or systemic dose effects (ILIB—Intravascular Laser Irradiation of Blood). We can consider, as limitations of this study, the lack of comparisons with other photobiomodulation protocols that use only infrared (IR) or red (R) light irradiation in the bone regeneration process. Uniquely in our studies, we chose to use equipment that allows for combined emission (R + IR), enabling us to harness the beneficial effects of both types of light regarding analgesic, anti-inflammatory effects, collagen and elastin formation, and other bio-stimulatory and bio-modulatory effects of low-level laser photobiomodulation [68]. The absence of mechanical tests to corroborate the microtomographic and histomorphometric results can also be considered. Based on this fact, future studies, mainly in long bones such as the tibia and femur, emerge as new perspectives.

Constant studies in regenerative medicine will still occur, involving the synthesis, all bioactive properties, and their biomedical applications, including potentially therapeutic nanoparticles, for the treatment of numerous diseases that affect the health of people and animals [69].

## 4. Conclusions

In this preclinical study, we evaluated the association of the world’s only fully heterologous fibrin biopolymer with a commercially available nano hydroxyapatite for bone repair, combined with a photobiomodulation protocol utilizing both red and infrared lasers concurrently.

Nanohydroxyapatite, either alone or with the biopolymer, demonstrated osteoconductive properties without significant inflammatory reactions. However, the biopolymer alone, when combined with low-level laser photobiomodulation therapy, exhibited the highest percentage of new bone formation at the evaluated periods (14 and 42 days post-operatively). In addition, the collagen fibers and the new bone tissue formed were more organized and had a greater degree of maturation.

There is clinical promise for the use of this biopolymer in the field of bioengineering and tissue regeneration, following the completion of phase III clinical trials using the bio product for chronic venous ulcer treatment.

## Figures and Tables

**Table 1:** Parameters used in PBM.

Parameter	Unit/Description
Laser type	Red (R): Indium Gallium Aluminum Phosphide (InGaAlP) Infrared (IR): Gallium Arsenide Aluminum (GaAsAl) Manufacturer: Therapy EC, DMC^®^ Equipments, São Carlos, SP, Brazil
Output power	100 mW ± 20%
Wavelength	660 nm ± 10 nm (R) e 808 nm ± 10 nm (IR)
Power density	1.01 mW/cm^2^
Energy density	30.48 J/cm^2^
Beam area	0.0984 cm^2^
Energy	6 J (R + IR)
Beam Type	Positioned perpendicularly to the skull
Emission Mode	Continuous
Application Method	Single central point
Irradiation duration	30 s
Treatment time	Immediately after surgery and twice weekly on alternate days until euthanasia

## Data Availability

Data presented in this study are available on request from the corresponding author.
